# Influence of AFM Tip Temperature on THF Hydrate Stability: Theoretical Model and Numerical Simulation

**DOI:** 10.1155/2019/1694169

**Published:** 2019-10-17

**Authors:** Li Peng, Fulong Ning, Wei Li, Jiaxin Sun, Pinqiang Cao, Zhichao Liu, Jingyu Xie

**Affiliations:** ^1^Faculty of Engineering, China University of Geosciences, Wuhan 430074, China; ^2^Laboratory for Marine Mineral Resources, Qingdao National Laboratory for Marine Science and Technology, Qingdao 266237, China

## Abstract

Atomic force microscopy (AFM) indentation is widely used to determine mechanical parameters of various materials. However, AFM tip may lead to phase transition of the cold sample in the region of contact area. It is a long-standing challenge that low-temperature phase-change materials (e.g., ice and hydrate) are hardly characterized by AFM, especially for clathrate hydrates. Here, with theoretical analysis and numerical simulation, we investigated the temperature influence of AFM tip on the tetrahydrofuran (THF) hydrate stability. At first, a steady-state model of heat conduction was established between a v-shaped probe and THF hydrate sample. The temperature of the tip was estimated at different laser spot positions and laser intensities. Through numerical simulation, the heat loss by air convection is less than 1% of the total laser heat, and the influence of ambient air on the AFM probe temperature can be neglected. Meanwhile, the local temperature in the region of contact area was also calculated at the THF hydrate temperature of 0°C, -10°C, -20°C, and -30°C. We found out that the AFM tip causes the cold THF hydrate to melt. The thermal melting thickness decreases with the reduction of laser intensity and THF hydrate temperature. On the contrary, it is positively correlated with the thickness of liquid-like layer on THF hydrate surface and is also linearly increased with the contact radius. This indicates that the thermal melting continues as the press-in depth of the tip into THF hydrate increases. The local temperature rises when the tip touches the THF hydrate. It is easier for THF hydrate to be melted by an external pressure. In addition, the proposed model may be useful for guiding force tests on low-temperature phase-change materials by the AFM indentation.

## 1. Introduction

Gas hydrate is a kind of ice-like crystalline compound formed by water and gas (such as methane, ethane) at high pressure and low temperature [1]. It is a potential energy source [2] and mostly distributed in marine sediments [3]. One of the keys to the successful exploitation of gas hydrate is the mechanical stability of gas hydrate-bearing sediments and wellbore [4, 5]. The mechanical property of gas hydrates at micro- and nanoscale is very important to explain that of gas hydrate-bearing sediments. Compared with gas hydrate, the stability condition (or phase equilibrium conditions) of tetrahydrofuran (THF) hydrate is easier to be created in laboratory. So THF hydrate instead of gas hydrate is often used as the research object.

**Figure 1:**
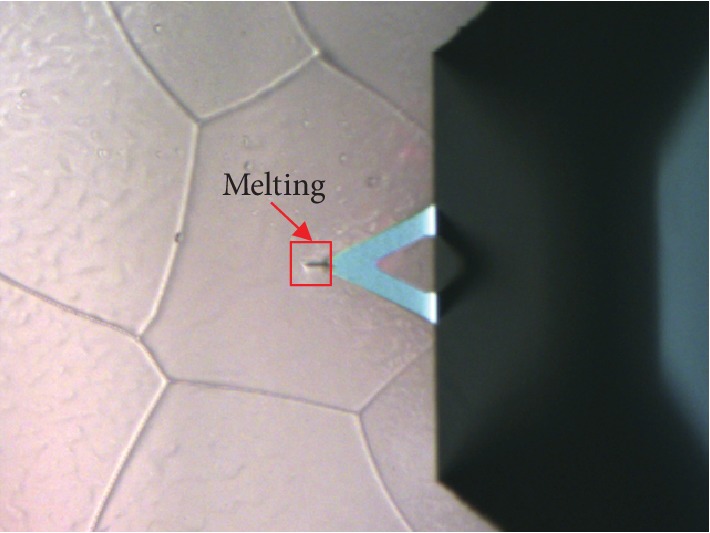
The melting THF hydrate under an AFM tip.

With atomic force microscopy (AFM), the micro- and nanomechanical properties of various materials are usually evaluated from force versus displacement curves [6]. In the AFM, the most common method for cantilever-deflection measurements is the beam-deflection method, in which laser beam from a solid-state diode is reflected by the back of the cantilever and collected by a position-sensitive detector (PSD). However, the laser beam may heat the probe cantilever, thereby increasing the temperature of the tip mounted on the end of the cantilever. Once the tip is in contact with the cold sample surfaces, the heat transferred from the tip will increase the local temperature of samples in the region of the contact area. For THF hydrate, of which phase equilibrium temperature is about 4.4°C [7], the variation in temperature may lead to inaccurate test results, and even to the failure of testing or sample damage due to the thermal melting of THF hydrate. As shown in Figure 1, there is an obvious scratch on THF hydrate surface although its temperature is -20°C. This scratch is likely caused by THF hydrate melting. The melting may result from the temperature difference between the tip and THF hydrate. Therefore, it is crucial to investigate the tip temperature and the local temperature change of THF hydrate samples during AFM measurements. In previous researches, the following methods were adopted to measure cold ice samples: using a cold chamber or freezer [8], using a cold sample holder [9] and submerging samples in organic solvents [10]. This sample would melt more or less unless the tip temperature is lowered enough. To reduce the heating effect, researchers tried to reduce the laser intensity by an optical filter [8, 11]. At the same time, previous studies also calculated the temperature of an AFM tip and the temperature difference between the end of the tip and sample stage [12]. However, it is unclear how the laser beam affects the temperature of AFM tip. Moreover, there are few reports about the heat effect of the tip on the cold THF hydrate sample.

To analyze the effect of the tip temperature on THF hydrate, a steady-state thermal conduction model was established between a v-shaped probe and THF hydrate for AFM indentation measurement. The temperature of tip and the local temperature in the contact region are calculated theoretically under various THF hydrate temperatures. Furthermore, to study the effects of air convection, a series of numerical simulations using ANSYS were also performed. The effect of laser intensity, laser position, temperatures, contact radius, and liquid-like layer on the melting of THF hydrate was studied.

## 2. Thermal Conduction Model

### 2.1. Physical Model

The power *W* of the laser beam focused on the back of probe cantilever is about 1.0 mW [8]. The laser absorptivity *k* of the probe cantilever may be related to the type of AFM and the type of probe. For our experiment, the absorptivity is very difficult to measure, so we refer to a lot of published papers [11–13] and find that it have been measured with a bimaterial AFM cantilever, which is approximately 0.13 [14]. Therefore, the value of *k* = 0.1 is determined in our study. Since the size of the substrate is one to two orders of magnitude larger than the cantilever, the heat conducted from the cantilevers is transmitted quickly to the environment. The temperature of the substrate can be approximately equal to the room temperature. When the probe is suspended in the air, most of the heat transfers to the probe substrate by heat conduction and a bit of heat transfers to the air by natural convection and thermal radiation. For microstructures immersed in air, heat conduction is a dominant mode in heat transfer, which may be more important than natural convection [15]. The movement and vibration of the probe will aggravate the air disturbance around the probe, which increases the heat convection. This problem is difficult to solve by mathematical method, so we use numerical simulation method to study it. In addition, previous studies reported that the radiative heat loss from the microcantilever to environment is about only 0.5% of the total dissipation heat when the microcantilever is heated to 570 K [15]. Therefore, the radiative heat can be negligible in heat transfer from the cantilever to the surrounding air and its influence is not considered in this paper.

**Figure 2:**
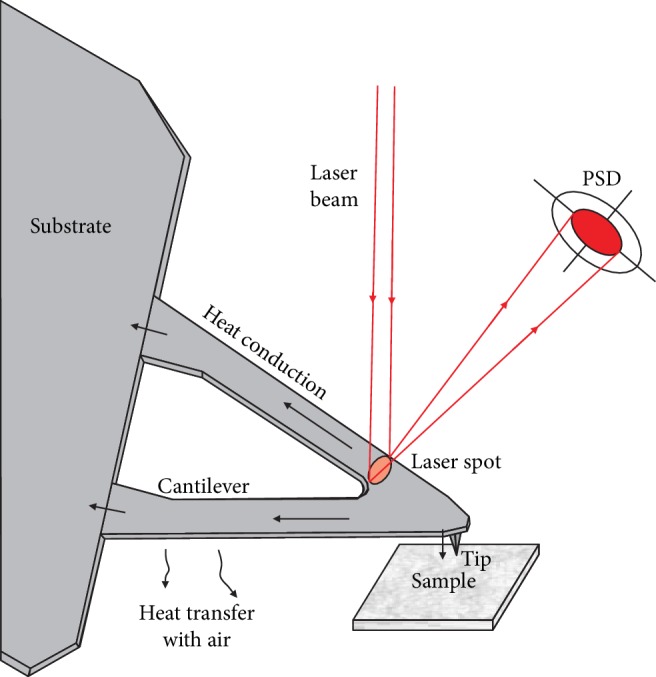
The heat conduction model between the probe and the sample.

When the tip is in contact with THF hydrate surfaces, little heat is transferred to the samples. The heat conduction model between the probe and the THF hydrate is shown in Figure 2. In this study, we assumed as follows: the probe and the THF hydrate are homogeneous and isotropous, the interface energy and thermal radiation between the tip and THF hydrates are ignored, and the THF hydrate surface is flat without considering its deformation.

**Figure 3:**
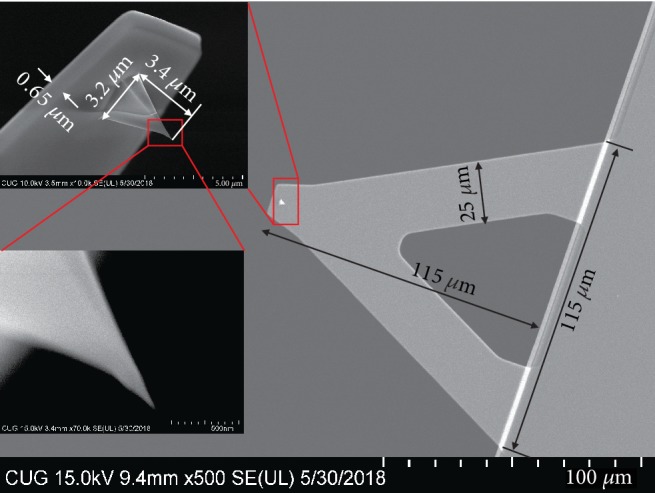
The shape and size of a selected AFM probe mapped by SEM.

The size of a v-shaped silicon nitride probe (SCANASYST-AIR) was measured by scanning electron microscopy, as shown in Figure 3. The probe cantilever has a length *L* of 115 *μ*m, a width *B* of 25 *μ*m, and a thickness *d* of 0.65 *μ*m. The tip is approximately a pyramid, 3.4 *μ*m in height *H*. The quadrilateral diagonal of the tip head is 3.2 *μ*m in length *a*. The radius of curvature of this tip is usually less than 12 nm.

### 2.2. Mathematical Model

**Figure 4:**
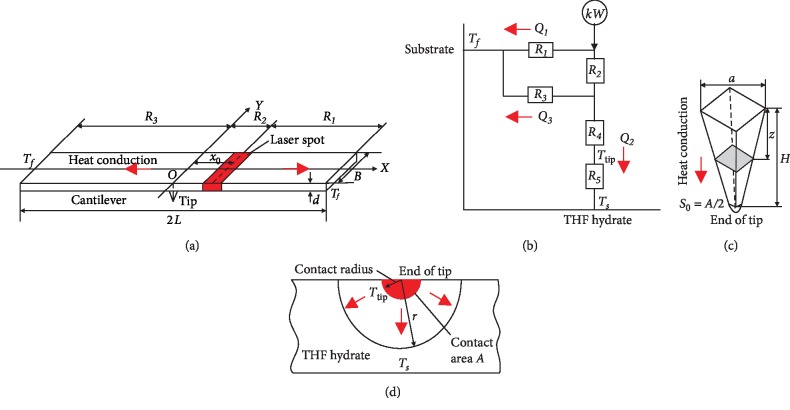
The heat transfer model between AFM probe and THF hydrate.

We supposed that the tip is at the origin of coordinates, *x*-axis is along the length of the probe cantilever and *y*-axis is along its width. Considering heat mainly transmits along the *x*-axis, as shown in Figure 4(a), the v-probed cantilever can be simplified to a long plate with a length of 2*L* and a width of *B*. The room temperature and sample temperature are expressed as *T*_*f*_ and *T*_*s*_, respectively. As shown in Figure 2, the laser beam focuses on *x* = *x*_0_. The simplified model is similar to electric circuit analog, as illustrated in Figure 4(b). There are three paths for heat transfer. As shown in Figure 4(a), *R*_1_ is the thermal resistance of cantilever between the area laser focused on and the probe substrate, *R*_2_ is the thermal resistance between the area laser focused on and the origin of coordinates, and *R*_3_ is the thermal resistance between the origin and the probe substrate. They can be expressed as follows:

(1) $$\begin{array}{c} R_{1}=\frac{L-x_{0}}{\lambda_{1}Bd}, \end{array}$$

(2) $$\begin{array}{c} R_{2}=\frac{x_{0}}{\lambda_{1}Bd}, \end{array}$$

(3) $$\begin{array}{c} R_{3}=\frac{L}{\lambda_{1}Bd}, \end{array}$$

where *λ*_1_ represents the thermal conductivity of probe material. In addition, the tip is assumed to be a right square pyramid and the end of the tip is considered as a hemisphere. The area of the cross section with a distance *z* to the square base can be described as a function *S*(*z*). The contact area *A* between the tip and the THF hydrate is shown in Figures 4(c) and 4(d). The thermal resistance *R*_4_ between the back of the cantilever and the end of the tip can be described as follows:

(4) $$\begin{array}{c} R_{4}=\int_{0}^{H}\frac{1}{\lambda_{1}S\left(z\right)}dz=\frac{2H}{a^{2}\lambda_{1}}\left(\frac{a}{\sqrt{2}A}+1\right). \end{array}$$

Because the contact area *A* between the tip and THF hydrate is as small as the nanometer scale, the tip can be regarded as a point heat source as shown in Figure 4(d). The thermal resistance *R*_5_ of the THF hydrate is as follows:

(5) $$\begin{array}{c} R_{5}=\frac{1}{2{\pi \lambda }_{2}}\left(\frac{1}{r_{0}}-\frac{1}{r_{1}}\right), \end{array}$$

where *λ*_2_ and *r*_0_ are the thermal conductivity of the THF hydrate and contact radius, respectively. In the region of contact area, the thermal conductivity of THF hydrate actually will change when its temperature increases. However, this variation in thermal conductivity is small [16]. Here, the thermal conductivity is considered as a constant. *r*_1_ is the length which is comparable to the size of a THF hydrate crystal. Since this length *r*_1_ is much larger than the contact radius *r*_0_, Equation (5) can be simplified as follows:

(6) $$\begin{array}{c} R_{5}=\frac{1}{\sqrt{2\pi A}\cdot \lambda_{2}}. \end{array}$$

The temperature of the contact area between the tip and the sample *T*_tip_ is as follows:

(7) $$\begin{array}{c} T_{tip}=T_{s}+R_{5}\cdot \frac{kWR_{1}R_{3}+\left(T_{f}-T_{s}\right)\left(R_{1}+R_{2}+R_{3}\right)}{\left(R_{4}+R_{5}\right)\left(R_{1}+R_{2}+R_{3}\right)+R_{3}\left(R_{1}+R_{2}\right)}. \end{array}$$

Substituting equations (1), (2), (3), (4), and (6) into equation (7), then *T*_tip_ is as follows:

(8) $$\begin{array}{c} T_{tip}=T_{s}+\frac{1}{\sqrt{2\pi A}\lambda_{2}}\cdot \frac{\left(L-x_{0}\right)kW+2\lambda_{1}Bd\left(T_{f}-T_{s}\right)}{2\lambda_{1}Bd\left[2H/a^{2}\lambda_{1}\left(\left(a/\sqrt{2A}\right)+\left(1\right)\right)+1/\sqrt{2\pi A}\lambda_{2}\right]+L}. \end{array}$$

The temperature at the distance *r* from the contact center *T*_s_(*r*) can be described as follows:

(9) $$\begin{array}{c} T_{s}\left(r\right)=\frac{T_{\text{tip}}-T_{s}}{r}\cdot \sqrt{\frac{A}{2\pi }}+T_{s}. \end{array}$$

If the tip does not contact the THF hydrate sample, the temperature of the tip is as follows:

(10) $$\begin{array}{c} T_{tip}=T_{f}+\frac{kWR_{1}\left(R_{2}+R_{3}\right)}{R_{1}+R_{2}+R_{3}}\cdot \frac{L}{L+x_{0}}=T_{f}+\frac{kW}{2\lambda_{1}Bd}\left(L-x_{0}\right). \end{array}$$

### 2.3. Numerical Method

**Figure 5:**
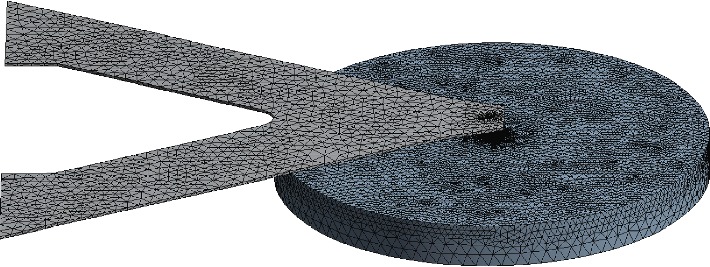
The mesh adopted in the numerical model.

According to the physical model and the SEM image of the probe, a numerical model was established by the finite element software, ANSYS. In this model, the THF hydrate is considered as a cylinder with a diameter of 100 *μ*m and a height of 10 *μ*m, whose top surface is perpendicular to the tip, as shown in Figure 5. The size of THF hydrate is about three orders of magnitude larger than that of the contact zone, so the periphery of THF hydrate is hardly influenced by the tip. The elements around the contact area between the tip and THF hydrate sample are refined (Figure 5).

There are three types of boundary conditions in the model. The first, temperatures of the probe substrate and at the side and bottom of THF hydrate remain unchanged, which are *T*_*f*_ and *T*_*s*_, respectively. The second, the power of heat induced by the laser beam is *kW*. The third, both the probe cantilevers and the upper surfaces of THF hydrate sample have heat exchange with the ambient air. In the case of numerical simulation, it is considered that the convective heat *q* between the probe and the air per unit area is the convective heat exchange coefficient *h* multiplied by their temperature difference *T*_probe_ − *T*_air_ [17, 18] which can be expressed as follows:

(11) $$\begin{array}{c} q=h\left(T_{\text{probe}}-T_{\text{air}}\right). \end{array}$$

*T*_probe_ is the local temperature of the probe and *T*_air_ is the air temperature. THF hydrate phase transition temperature is of approximate 4.4°C at atmospheric pressure. The thermal conductivity of THF hydrate sample varies with the temperature. The parameters of the numerical model are shown in Table 1.

## 3. Results and Discussion

### 3.1. The Temperature of the Tip

**Figure 6:**
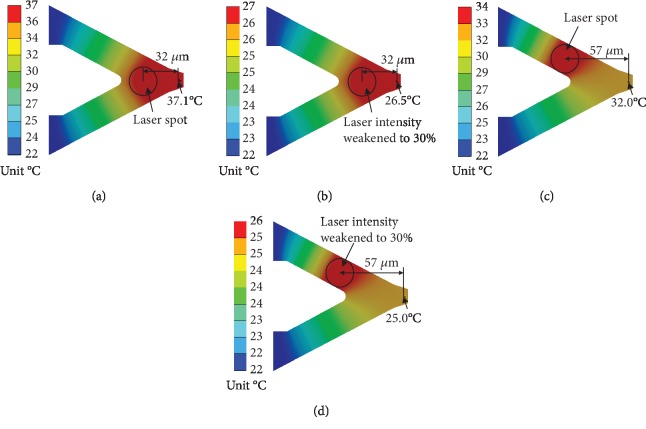
Temperature distribution of AFM probes heated by the laser beam. (a) The distance of 32 *μ*m between the laser spot and tip. (b) The laser intensity weakened to 30%. (c) The distance increases to 57 *μ*m. (d) The distance increases to 57 *μ*m and the laser intensity weakened to 30%.

Before the tip touches the THF hydrate, the probe temperature distribution at different laser intensities and positions on the cantilever is shown in Figure 6. It can be seen that the temperature of the cantilever linearly decreases with the distance from laser area to probe substrate. The temperature at the laser area is obviously higher than that of other area. As shown in Figure 6, when the distance between the laser spot and the tip is 32 *μ*m, the temperature of the tip is 37.1°C (Figure 6(a)). If the laser intensity is reduced to 30% of its original, the tip temperature will reduce to 26.5°C (Figure 6(b)). However, when the distance increases to 57 *μ*m, the tip temperature will reduce to 32.0°C (Figure 6(c)) or 25.0°C (Figure 6(d)) if we reduce the laser intensity to 30% at this distance. These results show that increasing the distance from the laser spot to the tip and lowering the laser intensity, the temperature of AFM tip will reduce.

The heat that transfers to the probe substrate, as in Figures 6(a)–6(d), are 9.949 × 10^−5^ W, 2.985 × 10^−5^ W, 9.958 × 10^−5^ W, and 2.9761 × 10^−5^ W, respectively. And the heat losses to ambient air are 5.097 × 10^−7^ W, 1.5292 × 10^−7^ W, 4.2105 × 10^−7^ W, and 1.2631 × 10^−7^ W accordingly. These results indicate that the heat transferred to ambient air is less than 1% of the total heat. In other word, the natural convection from the probe cantilever to surrounding air can be ignored, which is agree with the conclusions of the reference [19]. We compared the simulation results with the predictions by the mathematical model. According to equation (10), substituting *k* = 0.1, *W* = 0.001 W, *λ*_1_ = 18 W/m · °C, *B* = 25 *μ*m, *d* = 0.65 *μ*m, *L* = 115 *μ*m, *t*_*f*_ = 22°C, *T*_*f*_ = 22°C, *x*_0_ = 32 *μ*m, we obtain the temperature of the tip is 36.2°C under the laser condition as same as that shown in Figure 6(a). Furthermore, the predicted temperatures under the laser conditions as same as those show in Figures 6(b)–6(d) are 26.3°C, 31.9°C, and 25.0°C, respectively. The temperature difference by the two methods is 0~0.9°C, which is mainly caused by the simplified shape of the probe and neglected heat loss in the mathematical model.

### 3.2. Temperature Change of THF Hydrate

**Figure 7:**
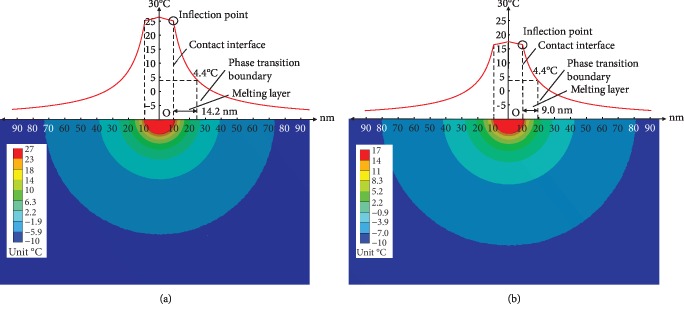
The numerical simulation results of the local temperature distribution around the contact interface, at conditions of the contact radius of 10 nm and THF hydrate temperature of -10°C. (a) The distance of 32 *μ*m between the laser spot and tip. (b) The distance increases to 57 *μ*m and the laser intensity weakened to 30%.

If the AFM tip touches the THF hydrate surface, the local temperature distribution around the contact interface as shown in Figure 7, it can be found that the temperature of the tip end is the highest in the contact region. And the temperature and the temperature gradient will decrease with the increase of the distance to the tip. However, there is an inflection point at contact interface between the tip and THF hydrate in temperature-distance curves (Figures 7(a) and 7(b)). This may be caused by the huge difference in thermal conductivity of these two different materials.

As shown in Figure 7(a), the temperature at contact interface is 25.1°C under the laser condition as same as that in Figure 6(a). The temperature will drop to -6.7°C at the distance 100 nm to the origin. Some THF hydrates near the tip will melt, because the temperature exceeds the phase transition temperature of 4.4°C. There is a interface between solid THF hydrate and melting liquid, namely, the phase transition boundary, where the temperature is 4.4°C. The distance between the phase transition boundary and contact interface is 14.2 nm (Figure 7(a)). It means that the thickness of the melting layer is 14.2 nm between the tip and solid THF hydrate.  Figure 7(b) shows the temperature distribution around the contact area at the laser condition as same as that shown in Figure 6(d). It can be seen that the temperature is 16.4°C at the contact interface and -7.3°C at a distance of 100 nm. The melting thickness is close to 9.0 nm. If the temperature of the tip end continues to increase, the melting area of THF hydrate will expand.

**Figure 8:**
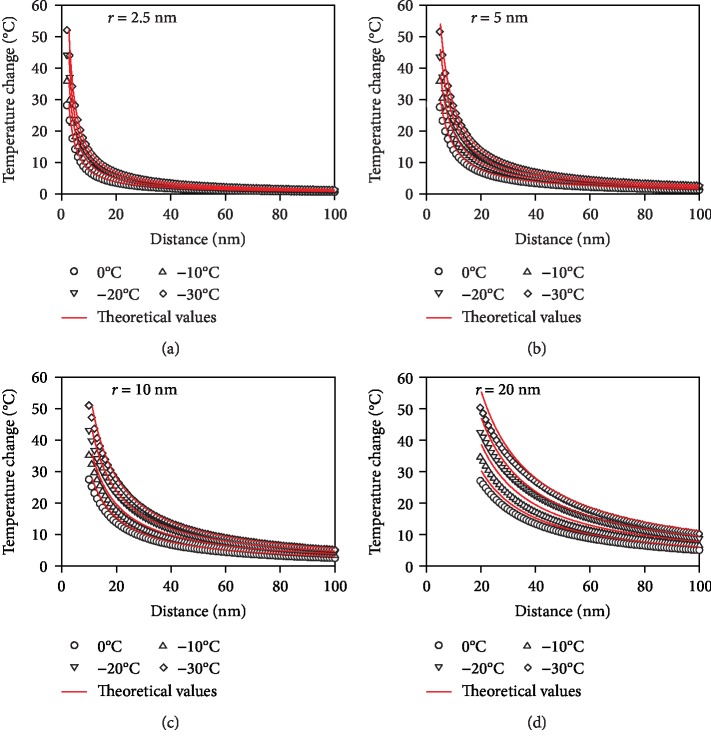
The local temperature change of THF hydrate at conditions that the laser spot is at distance of 32 *μ*m to the AFM tip and the contact radii are (a) *r*_0_ = 2.5 nm, (b) *r*_0_ = 5 nm, (c) *r*_0_ = 10 nm, and (d) *r*_0_ = 20 nm, respectively. The dots are obtained by numerical simulation and the red lines are from theoretical estimation.

Figure 8 illustrates the local temperature change of THF hydrate under different conditions (i.e., THF hydrate temperatures are 0°C, -10°C, -20°C, and -30°C, and the contact radii are 2.5 nm (Figure 8(a)), 5 nm (Figure 8(b)), 10 nm (Figure 8(c)), and 20 nm (Figure 8(d)). The numerical simulations take into consideration the dependence of thermal conductivity of THF hydrate samples on temperature, but the theoretical calculation does not consider (i.e., *λ*_2_ = 0.584 W/m · °C). This cause the theoretical values are slightly higher than those of the numerical simulations. The smaller the distance to tip is, the more noticeable difference between the two methods is, as shown in Figure 8. As the distance to the tip gradually increases, it is found that the temperature change of the THF hydrate decreases sharply at first, and then gradually slows down until the temperature change closes to zero. At the same distance to the tip, the lower temperature of THF hydrate, the more obvious the temperature change is. A larger contact radius has a greater influence on local temperature of THF hydrate. When the contact radius is less than 5 nm, the radius of the region is about 100 nm, where the temperature increases significantly (Figure 8(b)).

**Figure 9:**
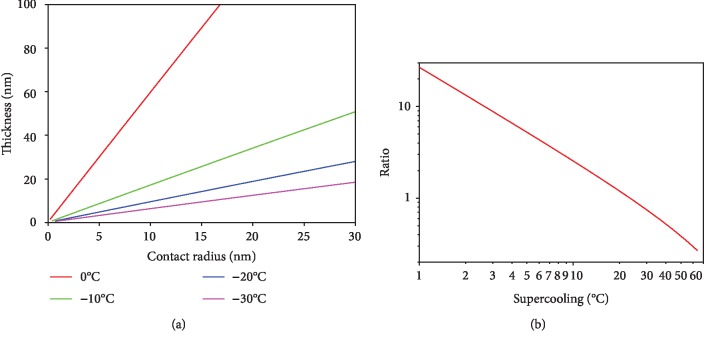
(a) The melting layer thickness. (b) Ratios of the thickness to contact radius at conditions that the laser spot is at distance of 32 *μ*m to the tip.

Figure 9(a) shows the relationship between THF hydrate melting layer thickness and the contact radius at the temperatures of 0°C, -10°C, -20°C, and -30°C. With the increase of the contact radius, the thickness increases linearly. If THF hydrate temperature lowers from 0°C to -10°C, -20°C, and -30°C, the line's slopes also reduce from 5.99 to 1.72, 0.96, and 0.63, respectively. This indicates that reducing sample temperature can effectively weaken its heat melting.  Figure 9(b) illustrates the relationship between the supercooling and the ratio of the melting layer thickness to the contact radius. This supercooling is defined as the difference between the phase transition temperature of THF hydrate and its actual temperature, which can denote stability degree of THF hydrate. There is a linear relationship between the logarithm of the ratio and the logarithm of the supercooling.

### 3.3. Temperature Influence on the Pressure-Induced Melting

**Figure 10:**
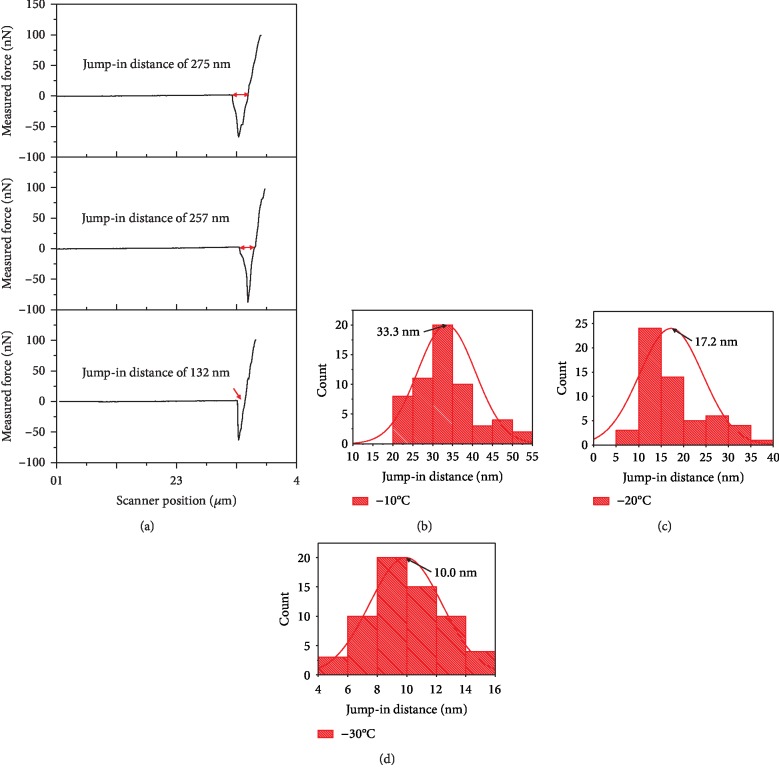
Atomic force microscopy (Bruker, Dimension Edge) was used to study melting of THF hydrate. The probe spring constant is about 0.4 N/m. The atomic force microscopy is placed in a glove box, in which the dew point of the air is below -35°C. THF hydrate was prepared and tested on a cold table. (a) Approaching part of force-displacement curves measured at the THF hydrate temperature of -20°C. After the laser intensity decreases to 30% with an optical filter, the jump-in distances on THF hydrate were determined from force-displacement curves, at the temperatures of -10°C (b), -20°C (c), and -30°C (d).

We used AFM to determine the jump-in distance on THF hydrate. At THF hydrate of -20°C, the jump-in distance from each test is quite different, as shown in Figure 10(a). The jump-in distances obtained from the three tests are 132 nm, 257 nm, and 275 nm, respectively. When the laser intensity of AFM is reduced to 30% with an optical filter, the average distances measured at THF hydrate temperatures of -10°C, -20°C, and -30°C are about 33.3 nm, 17.2 nm, and 10.0 nm, respectively (Figures 10(b)–10(d)). These jump-in distances and its trend changing with temperature are in agreement with those reported by references [9, 11] for ice.

When the tip first contacts the THF hydrate surface during an approach, the jump-in will occur if the force gradient exerted on the tip is greater than the spring constant of AFM probe. Once the tip touches the surface of the liquid-like layer (LLL) [20, 21], the tip surface is wetted by the liquid [13]. The capillary force is produced on the bending of the liquid surface at the meniscus [13]. This capillary force can pull the tip in the THF hydrate and the probe cantilever deflects downward. If the tip is moved toward the solid THF hydrate surface, the repulsive force exerted on the tip increases gradually. When the repulsive force equals the capillary force, the probe cantilever straightens. The displacement of the tip, beginning with the cantilever deflecting downward and ending with the cantilever straightening, is equal to the jump-in distance. Comparing the results of Figure 10(a) and Figure 10(c), we found that the significant decrease in jump-in distance is related to the decrease in tip temperature. When the laser intensity decreases to 30%, the tip temperature decreases and the melting layer of THF hydrate becomes thin (Figure 7). The melting of THF hydrate reduces the tip resistance. The melted liquid will be extruded from the gap between the tip and the solid THF hydrate. The wetted perimeter of the liquid on the tip surface will increase, resulting in the capillary force increasing [22, 23]. The reduced resistance and the increased capillary force will increase the depth of the tip indentation and the jump-in distance on the THF hydrate surface.

Thermal melting or pressure melting is likely to occur when the tip touches the solid THF hydrate. Despite the THF hydrate-tip, tip-LLL, and LLL-THF hydrate interface changes, the effect of interface free energy will be reduced if a melting layer is formed between the tip and the solid THF hydrate. Conversely, the melting of THF increases the contact radius which promotes thermal melting further, as shown in Figures 8 and 9(a). In addition, the pressure melting will happen only when the pressure exerted on solid THF hydrate exceeds a certain threshold. This threshold decreases with the increase of temperature [24]. The local temperature of THF hydrate rises due to contact with the tip, which makes it easier to melt by the external pressure.

### 3.4. Influence of Liquid-Like Layer on THF Hydrate Surface

**Figure 11:**
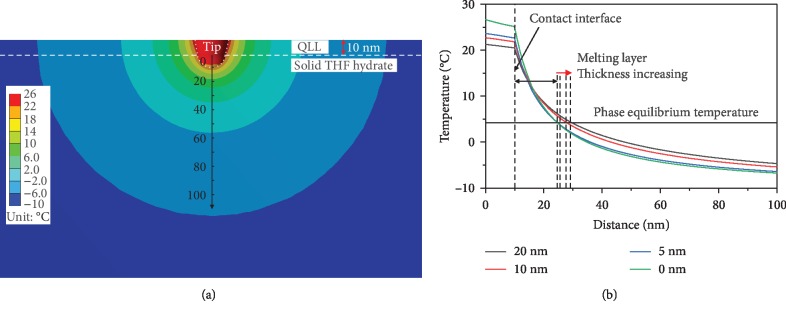
(a) The local temperature distribution around the contact interface, at conditions of the contact radius of 10 nm, THF hydrate temperature of -10°C, the distance of 32 *μ*m between the laser spot and tip, and the thickness of liquid-like layer of 10 nm. (b) The temperature vs. distance curves with the liquid-like layer thickness of 0 nm, 5 nm, 10 nm, and 20 nm.

As discussed above, the thickness of liquid-like layer on THF hydrate surface is less than the jump-in distance determined from force-displacement curves. So it is less than 33.3 nm at -10°C (Figure 10(b)), which is very close to the results tested on ice surface [11]. When the tip passes through the liquid-like layer and contacts with the solid THF hydrate, some heat transfers from the tip to solid THF hydrate and the liquid-like layer. The heat transferred to the liquid-like layer is eventually transferred to the solid THF hydrate. Given the contact radius between tip and solid THF hydrate, the heat conduction area between tip and hydrate sample increases due to the existence of liquid-like layer. This leads to an increase in the heat eventually transferred to the solid hydrate.  Figure 11(a) gives the numerical simulation results of the local temperature of contact area, at the conditions of liquid-like layer thickness of 10 nm, sample temperature of -10°C, and contact radius of 10 nm. Compared with Figure 7(a), the temperature rising area in Figure 11(a) is larger.  Figure 11(b) shows the temperature change from the tip to THF hydrate, under the conditions that the thickness of liquid-like layer changes from 0 to 5, 10, and 20 nm. Temperature of the tip decreases with the increase of the liquid-like layer thickness. If the thickness of liquid-like layer ranges from 0 to 20 nm, the melting layer thickness increases from 14.2 to 18.5 nm between the tip and solid THF hydrate. These indicate that the liquid-like layer on THF hydrate surface makes the temperature rising area and the melting layer thickness large.

## 4. Conclusions

A steady-state analytical model of thermal conduction was established between a v-shaped AFM probe and THF hydrate sample. The temperature of the tip was estimated at different laser spot positions and laser intensities. The laser intensity and the laser spot position on a probe cantilever have an obvious influence on the temperature of AFM tip. Through numerical simulation, the heat loss by air convection is less than 1% of the total laser heat, and the influence of ambient air on the AFM probe temperature can be neglected.

The local temperature in the region of contact area was also calculated at THF hydrate temperature of 0°C, -10°C, -20°C, and -30°C. The temperature of THF hydrate near the tip exceeds 4.4°C (phase equilibrium temperature of THF hydrate at atmospheric pressure), which means that the tip of AFM leads to the thermal melting of THF hydrate. There is a melting layer between the tip and the solid THF hydrate. The thermal melting layer thickness decreases with the reduction of laser intensity and THF hydrate temperature. On the contrary, the thickness is positively correlated with the thickness of liquid-like layer on THF hydrate surface. And it is linearly increased with the contact radius. This indicates that the thermal melting continues as the press-in depth of the tip into THF hydrate increases. The local temperature rises when the tip touches the THF hydrate. It is easier for THF hydrate to be melted by the external pressure. For AFM indentation measurement on the cold clathrate hydrates, the thermal melting needs to be considered unless the temperature of the tip is lowered enough.

## Figures and Tables

**Table 1:** The parameters of the numerical model [12, 16].

Parameter name	Value	Units
Power of the laser beam, *W*	0.001	W
Laser absorptivity, *k*	0.1	
Convective heat exchange coefficient, *h*	13.2	W/m^2^·°C
Room temperature, *T*_*f*_	22	°C
Thermal conductivity of the N_4_Si_3_ probe, *λ*_1_	18	W/m·°C
Thermal conductivity of THF hydrate sample, *λ*_2_	0.489 (≤−25°C), 0.496 ( = −7.5°C), 0.58 ( = 3.0°C), 0.584 (≥4.4°C) Linear interpolation (others)	W/m·°C
THF hydrate temperature, *T*_*s*_	-30, -20, -10, 0	°C
Contact radii, *r*_0_	2.5, 5, 10, 20	nm

## Data Availability

No data were used to support this study.
